# Evaluation of early treatment with intravenous idursulfase and intrathecal idursulfase‐IT on cognitive function in siblings with neuronopathic mucopolysaccharidosis II

**DOI:** 10.1002/jimd.12790

**Published:** 2024-09-09

**Authors:** Joseph Muenzer, Barbara K. Burton, Paul Harmatz, Luis González Gutiérrez‐Solana, Matilde Ruiz‐Garcia, Simon A. Jones, Nathalie Guffon, Michal Inbar‐Feigenberg, Drago Bratkovic, Stewart Rust, Michael Hale, Yuna Wu, Karen S. Yee, David A. H. Whiteman, David Alexanderian

**Affiliations:** ^1^ University of North Carolina at Chapel Hill Chapel Hill North Carolina USA; ^2^ Ann & Robert H. Lurie Children's Hospital of Chicago Northwestern University Chicago Illinois USA; ^3^ UCSF Benioff Children's Hospital Oakland Oakland California USA; ^4^ Infant Jesus Children's Hospital Madrid Spain; ^5^ National Institute of Pediatrics Mexico City Mexico; ^6^ St Mary's Hospital, Manchester University NHS Foundation Trust University of Manchester Manchester UK; ^7^ Reference Centre for Inherited Metabolic Disorder Hospices Civils de Lyon Lyon France; ^8^ University of Toronto Toronto Ontario Canada; ^9^ The Hospital for Sick Children Toronto Ontario Canada; ^10^ Women's and Children's Hospital North Adelaide South Australia Australia; ^11^ Manchester University NHS Foundation Trust Manchester UK; ^12^ Takeda Development Center Americas, Inc. Cambridge Massachusetts USA; ^13^ Hale Scientific Statistics, LLC Beaverton Oregon USA; ^14^ Takeda Development Center Americas, Inc. Lexington Massachusetts USA; ^15^ Present address: Alexion Pharmaceuticals, Inc., AstraZeneca Rare Disease Boston Massachusetts USA; ^16^ Present address: Merck Boston Massachusetts USA

**Keywords:** cognitive function, enzyme replacement therapy, idursulfase, intrathecal, neuronopathic mucopolysaccharidosis II, siblings

## Abstract

Mucopolysaccharidosis II (MPS II; Hunter syndrome; OMIM 309900) is a rare, X‐linked, heterogeneous lysosomal storage disease. Approximately two‐thirds of patients develop cognitive impairment, which is difficult to assess in clinical trials, partly owing to the variable nature of cognitive impairment. Analyzing data from siblings can help to minimize this heterogeneity. We report analyses of cognitive function from siblings with MPS II enrolled in clinical trials: a natural history study (NCT01822184), a randomized, open‐label, phase 2/3 study of intravenous (IV) idursulfase with or without intrathecal idursulfase (idursulfase‐IT; NCT02055118), and its extension (NCT2412787). Cognitive function was assessed using Differential Abilities Scales, Second Edition General Conceptual Ability (DAS‐II GCA) scores; Bayley Scales of Infant and Toddler Development, Third Edition; and Vineland Adaptive Behavior Scales, Second Edition Adaptive Behavior Composite (VABS‐II ABC). Seven sets of siblings (six pairs and one set of three) were included. All patients received IV idursulfase and 10 received subsequent idursulfase‐IT. Younger siblings initiated IV idursulfase at an earlier age than their older sibling(s) in six of the sets; the younger sibling started treatment before 1 year of age in three sets. Monthly idursulfase‐IT was generally associated with a stabilization of cognitive function: DAS‐II GCA and VABS‐II ABC scores were higher at age‐matched assessments in the majority of those who either received idursulfase‐IT earlier than their sibling or who received idursulfase‐IT versus no idursulfase‐IT. These data suggest that early initiation of intrathecal enzyme replacement therapy may stabilize or slow cognitive decline in some patients with neuronopathic MPS II.

## 1 INTRODUCTION

Mucopolysaccharidosis II (MPS II; Hunter syndrome; OMIM 309900) is a rare, X‐linked, lysosomal storage disease caused by deficient activity of iduronate‐2‐sulfatase (I2S) and subsequent lysosomal accumulation of glycosaminoglycans in multiple organs.^1^, ^2^ Somatic manifestations are present in all patients with MPS II and include facial abnormalities, short stature, cardiovascular disease, respiratory disease, and impaired joint mobility.^1^, ^2^, ^3^, ^4^ Approximately two‐thirds of patients with MPS II have the neuronopathic form of the disease and develop cognitive impairment.^4^ Although patients with non‐neuronopathic MPS II may survive into adulthood without treatment, those with neuronopathic MPS II typically survive only until the first or second decade of life.^4^

The current standard of care for both forms of MPS II is intravenous (IV) enzyme replacement therapy (ERT) with recombinant I2S (idursulfase [marketed as Elaprase®]; Takeda Pharmaceuticals USA, Inc., Lexington, MA, USA).^5^, ^6^ Weekly IV idursulfase (0.5 mg/kg) treatment improves or stabilizes some of the somatic manifestations of MPS II but has not been shown to cross the blood–brain barrier at sufficient concentrations to slow cognitive decline.^7^, ^8^ A formulation of idursulfase for intrathecal (IT) administration (idursulfase‐IT) has been developed to treat the neurological manifestations of MPS II.^9^ The efficacy and safety of idursulfase‐IT were assessed in a phase 2/3 randomized, open‐label study in patients aged 3 years to younger than 18 years, and in a single‐arm, open‐label substudy in those younger than 3 years.^10^, ^11^, ^12^ Patients who completed either study were enrolled in an ongoing open‐label extension, in which all patients received idursulfase‐IT.^10^, ^11^, ^12^ Although the primary endpoint of the phase 2/3 study (change from baseline in Differential Abilities Scales, Second Edition General Conceptual Ability [DAS‐II GCA] score at week 52) was not met,^10^ post hoc analyses indicated a trend towards potential cognitive benefits of idursulfase‐IT versus no idursulfase‐IT for the subgroup of patients with missense iduronate‐2‐sulfatase gene (*IDS*) variants who started idursulfase‐IT before 6 years of age.^10^

Potential effects of early treatment initiation on cognitive function can be challenging to assess in clinical trials, especially in diseases such as MPS II with variable onset and rates of disease progression.^13^ Clinical variability can be seen even among patients with the same variant,^13^ and factors such as hearing loss can influence neurological testing if not appropriately accounted for.^14^ Tools used to evaluate cognitive function need to have tests at the appropriate difficulty level, be sensitive to disease‐specific progression, and have appropriate normative data for comparison.^15^ One approach that can provide additional insights into cognitive function in patients with MPS II is to analyze data from siblings. Siblings have a shared genotype and are more likely to have shared a similar environment, reducing heterogeneity.^16^ Detailed information on one sibling set from the phase 2/3 study has been published recently.^17^ To further explore cognitive function in siblings with neuronopathic MPS II, we evaluated cognitive function changes over time in all sibling sets enrolled in a natural history study and/or idursulfase‐IT clinical trials for whom age‐matched data were available.

## 2 METHODS

### 2.1 Study designs and patients

Sibling sets were identified by the study sites from among patients participating in at least one of the studies described below. All siblings were required to have participated in at least one of the studies to be eligible for inclusion in these analyses. In all studies, patients received weekly IV idursulfase as standard of care.^10^, ^11^, ^12^, ^18^

The natural history study (NCT01822184) was an observational, prospective, longitudinal study that assessed cognitive status and adaptive behavior for up to 2 years, in 55 male patients with MPS II who were aged 2 to younger than 18 years and had a DAS‐II GCA score above 55. Patients were not required to have cognitive impairment and eligible patients from this study could enroll in the phase 2/3 study.^18^

The primary phase 2/3 study was a 52‐week, randomized controlled trial (NCT02055118) in which eligible patients were randomized 2:1 to receive idursulfase‐IT 10 mg once every 28 days or no idursulfase‐IT. All patients received IV idursulfase 0.5 mg/kg once a week as standard of care.^10^ IV idursulfase was administered at least 48 h after idursulfase‐IT. The phase 2/3 primary study assessed the efficacy and safety of idursulfase‐IT in males with MPS II aged 3 to younger than 18 years with a documented diagnosis of MPS II, and evidence of cognitive impairment (as determined by DAS‐II GCA scores).^10^

A substudy of the 52‐week trial was undertaken in patients younger than 3 years of age at baseline. This was a non‐randomized, open‐label, single‐arm, 52‐week study in which all patients received treatment with idursulfase‐IT (dose was adjusted according to age as follows: 5 mg for patients up to 8 months of age at dosing; 7.5 mg for patients aged 8 months to younger than 30 months at dosing; and 10 mg for patients older than 30 months at dosing).^12^

In the ongoing, open‐label extension study (NCT02412787) and the associated substudy, all patients receive idursulfase‐IT 10 mg once every 28 days, except those aged 8 months to younger than 30 months at dosing, who instead receive idursulfase‐IT 7.5 mg once every 28 days.^11^

The design of these studies is described in more detail separately; studies were conducted in accordance with the Declaration of Helsinki and written informed consent was obtained.^10^, ^11^, ^12^, ^18^

### 2.2 Assessments

Cognitive function was assessed using DAS‐II GCA and the Vineland Adaptive Behavior Scales, Second Edition Adaptive Behavior Composite (VABS‐II ABC).^19^, ^20^, ^21^, ^22^ In this study, the DAS‐II GCA comprised an early‐years battery for children aged 2 years 6 months to 6 years 11 months, and a school‐age battery for those aged 7 years to 17 years 11 months.^10^ The DAS‐II GCA score measures cognitive ability relative to a same‐aged normative sample; the mean score is 100, with a standard deviation (SD) of 15. In the substudy, the Bayley Scales of Infant and Toddler Development, Third Edition (BSID‐III) was initially used to assess cognitive function in patients younger than 42 months of age; assessment was transitioned to the DAS‐II GCA at 42 months of age, if appropriate. The BSID‐III score consists of five subtests (cognitive, language, motor, social–emotional, and adaptive) that are converted to composite scores. Similar to DAS‐II GCA, BSID‐III is relative to a same‐aged normative sample; the mean score is 100, with a SD of 15.^23^ VABS‐II assesses adaptive behaviors of individuals from birth to 90 years 11 months of age. The VABS‐II ABC is a composite score of the four VABS‐II domains (communication, daily living, socialization, and motor skills) in patients aged younger than 7 years and a composite of communication, daily living, and socialization skills in patients aged 7 years or older. The ABC and domains return age‐based standard scores with a normative mean of 100 and a SD of 15, and subdomains return standard scores with a mean of 15 and a SD of 3.^21^, ^22^ Full details on the assessment methodology within the included studies are published elsewhere.^10^, ^11^, ^12^, ^18^

Individual DAS‐II GCA early‐years and school‐age battery scores by chronological age were plotted, and age‐matched DAS‐II GCA scores were compared between siblings. Similarly, individual VABS‐II ABC scores were plotted, and age‐matched VABS‐II were compared between siblings.

### 2.3 Statistical analyses

Data from the primary phase 2/3 study, extension and substudy were included. No formal statistical analysis was performed. The data cut‐off date was July 30, 2019, which was selected for consistency with the individual studies' pre‐determined interim analyses dates.

## 3 RESULTS

### 3.1 Patients

In total, seven sets of siblings comprising 15 patients (six pairs and one set of three) were analyzed. One additional sibling pair (with both patients enrolled only in the natural history study) was identified but was not included in this analysis because neither sibling received idursulfase‐IT and age‐matched data were not available (Table S1; Figure S1). These analyses do not include data from patients for which only one of the siblings was included in a study.

**FIGURE 1:**
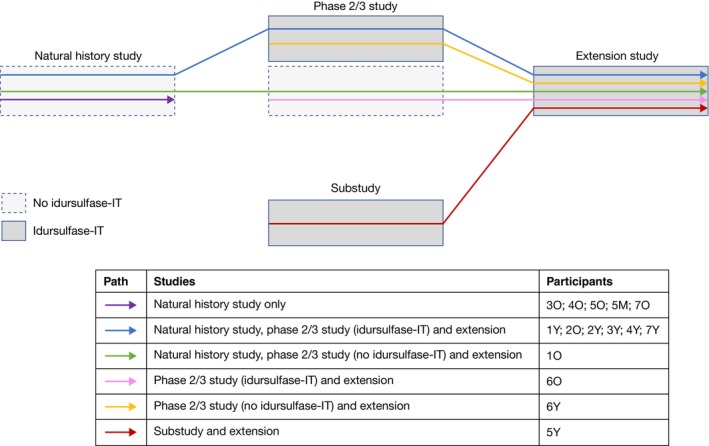
Patient flow and treatment pattern. All patients received weekly intravenous idursulfase as standard of care in each study. Arrows represent participation, and do not necessarily imply completion of a study. Patient coding refers to sibling group number (1–7) and relative age (Y, youngest; M, middle; O, oldest).

The patient flow for the four contributing studies is illustrated in Figure 1. Most of the patients (80.0%; 12/15) participated in the natural history study and the majority of these (58.0%; 7/12) went on to participate in the phase 2/3 study and its associated extension. Age‐matched data were available for six sets of siblings (sets 1–6). In set 7, the older sibling (aged 6 years) was screened for the phase 2/3 study but was unable to perform the DAS‐II GCA tasks and therefore did not meet the eligibility criteria; no DAS‐II GCA data are available for this patient.

Baseline characteristics for all 15 patients are shown in Table 1; patients were aged 2.0–6.5 years and genotypes were the same for each sibling set. No formal inclusion criteria were applied for genotype, however, all patients had missense *IDS* variants. Genotype data were not available for one patient and it was assumed that this individual had the same genotype as their sibling. All patients received IV idursulfase, which was initiated at age 0.1–4.6 years. Five patients (33.3%) participated only in the natural history study and so did not receive idursulfase‐IT treatment. Of the 10 patients (66.7%) who also received idursulfase‐IT treatment, seven received idursulfase‐IT during the phase 2/3 study and its associated extension; one received idursulfase‐IT in the substudy and its extension, and two received idursulfase‐IT in the extension only, after receiving only IV idursulfase in the primary study. Age at idursulfase‐IT treatment initiation ranged from 3.0 to 8.8 years.

**TABLE 1:** Baseline characteristics and treatment status.

	Set 1		Set 2		Set 3		Set 4		Set 5			Set 6		Set 7	
	Y	O	Y	O	Y	O	Y	O	Y	M	O	Y	O	Y	O
*Patient characteristics*															
Age, years^a^	4.0	6.5	3.3	5.1	2.0	5.2	2.3	4.3	3.0	3.7	4.9	3.9	5.1	3.5	6
Genotype	C.134A>G (P.D45G)		C.1504 T>G (P.W502G)		C.998C>T (P.S333L)		C.283A>G (P.R95G)		C.257C>T (P.P86L)			C.1402C>T (P.R468W)		C.1400C> T (P.P467L)	Unknown
Earliest available DAS‐II GCA score^a^	96	60	90	56	93^b^	54^c^	84^c^	40^c^	80^d^	70	59	75	56	73	–
Earliest available VABS‐II ABC score	101	74	88	76	74	75	104	57	103	66	67	81	79	70	–
Age at earliest VABS‐II assessment, years	4.0	6.5	3.3	5.1	2.0	5.2	2.3	4.3	3.0	3.7	4.9	3.9	5.1	3.5	–
*Treatment status*															
Age at initiation of IV idursulfase, years^e^	2.1	4.6	3.1	3.8	0.3	3.2	0.1	1.8	0.1	1.5	2.7	1.8	3.1	2.9	–
Age at initiation of idursulfase‐IT, years	5.7	8.8	5.4	6.4	4.4	No IT treatment	3.9	No IT treatment	3.0	No IT treatment	No IT treatment	4.9	5.1	4.8	No IT treatment

Abbreviations: BSID‐III, Bayley Scales of Infant and Toddler Development, Third Edition; DAS‐II GCA, Differential Abilities Scales, Second Edition General Conceptual Ability; IT, intrathecal; IV, intravenous; M, middle sibling; O, oldest sibling; VABS‐II ABC, Vineland Adaptive Behavior Scales, Second Edition Adaptive Behavior Composite; Y, youngest sibling.

^a^ Data collected at screening in the natural history study or for patients who did not participate in the natural history study, data collected at baseline in the phase 2/3 trial or its associated substudy.

^b^ Data collected at month 9 in the natural history study.

^c^ Data collected at month 3 in the natural history study.

^d^ Data collected at week 40 in the phase 2/3 trial (cognitive function was previously assessed using the BSID‐III in the substudy before assessment was transitioned to the DAS‐II).

^e^ Data extracted from the concomitant medicine case report form and thus may be an approximate age.

### 3.2 DAS‐II GCA scores

**FIGURE 2:**
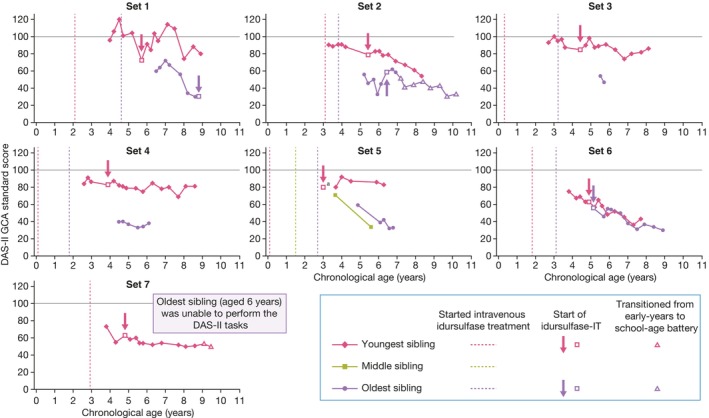
Individual profile plots of DAS‐II GCA early‐years and school‐age battery standard scores by chronological age for the seven sibling sets. ^a^The youngest sibling was enrolled in the substudy where they received idursulfase‐IT; cognitive function was initially assessed using BSID‐III before assessment was transitioned to DAS‐II GCA (BSID‐III data not shown). BSID‐III, Bayley Scales of Infant and Toddler Development, Third Edition; DAS‐II GCA, Differential Abilities Scales, Second Edition General Conceptual Ability; IT, intrathecal.

For the 14 patients who met eligibility criteria across seven sibling sets, the earliest available DAS‐II GCA scores ranged from 40 to 96 (Table 1), and patient ages at the time of the earliest available DAS‐II GCA scores were between 2.6 and 6.5 years. In sibling sets 1–5, the youngest sibling had a higher DAS‐II GCA score than the older sibling(s) in the age‐matched assessments (Figure 2). In three of these sets (sets 3–5), the youngest sibling received IV idursulfase before 1 year of age (Table 1). In set 6, both siblings started idursulfase‐IT at a similar age (4.9 and 5.1 years) and had similar DAS‐II GCA scores at age‐matched assessments (Figure 2). In set 7, the older sibling was unable to perform the DAS‐II GCA tasks. For two patients (the older sibling in set 2 and the younger sibling in set 7), DAS‐II GCA was initially assessed with the early‐years battery before transitioning to the school‐age battery; 12 patients were assessed with the early‐years battery throughout. One patient (the older sibling in set 2) who initiated idursulfase‐IT at 6.4 years, transitioned to the school‐age battery aged 7.2 years, and one patient (the younger sibling in set 7) who initiated idursulfase at 4.8 years, transitioned to the school‐age battery aged 9.1 years.

DAS‐II GCA scores were generally higher at age‐matched assessments in siblings who started IV idursulfase at a younger age than their sibling(s), as demonstrated by sets 1–5 (Figure 2). A similar pattern for DAS‐II GCA scores was observed in younger siblings who received idursulfase‐IT treatment earlier than their older sibling (sets 1 and 2), and in those who received idursulfase‐IT compared with their siblings who did not (sets 3–5). In set 1, for example, the eldest sibling received idursulfase‐IT at 8.8 years of age and had a DAS‐II GCA early‐years battery score of 30 at this timepoint, whereas the younger sibling who started idursulfase‐IT at age 5.7 years had a DAS‐II GCA early‐years battery score of 80 at age 8.9 years. Since 13 of 14 patients received IV idursulfase before the age of 4 years and only two patients received idursulfase‐IT before the age of 4 years, comparisons between the effects of early IV and early IT treatment cannot be made.

### 3.3 BSID‐III scores

In the substudy, cognitive function was assessed using the BSID‐III before assessment could transition to the DAS‐II (at 42 months of age). Consequently, only one patient (the youngest sibling in set 5) had BSID‐III data available; the earliest available BSID‐III cognitive composite score was 80 (at age 3.0 years), which increased to 100 (at age 3.5 years). IV idursulfase was initiated in this patient at age 0.1 years and idursulfase‐IT was initiated when the patient entered the phase 2/3 study at age 3.0 years. No comparisons or conclusions can be drawn based on the isolation of BSID‐III data in this patient, especially given the patient's young age and short follow‐up time. It is possible that any potential decline in cognitive function had not yet manifested for this patient, or that the progression of MPS II may be slow in this patient.

### 3.4 VABS‐II scores

**FIGURE 3:**
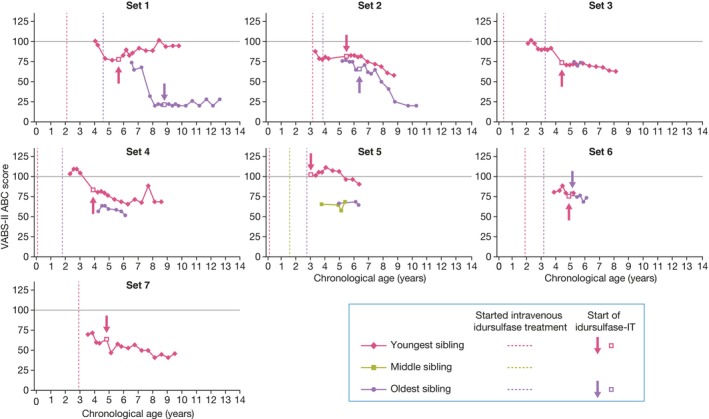
Individual profile plots of VABS‐II ABC scores by chronological age for the seven sibling sets. IT, intrathecal; VABS‐II ABC, Vineland Adaptive Behavior Scales, Second Edition Adaptive Behavior Composite.

For the 14 patients across seven sibling sets, the earliest available VABS‐II ABC scores ranged from 57 to 104 (Table 1), and patient ages at the time of earliest available VABS‐II ABC scores were between 2.3 and 6.5 years. In sets 1, 2, 4, and 5, the youngest sibling had a higher VABS‐II ABC score than the older sibling(s) at the same chronological age (Figure 3). In sets 3 and 6, VABS‐II ABC scores were similar between siblings at the same chronological age. As with the DAS‐II GCA assessments, the older sibling in set 7 was unable to perform the tasks.

Similar to the pattern observed with the DAS‐II GCA scores, VABS‐II ABC scores were generally higher at age‐matched assessments in the majority of siblings who initiated IV idursulfase at a younger age, particularly in sets 1 and 5 (Figure 3). A similar pattern for VABS‐II ABC scores was observed either for younger siblings who received idursulfase‐IT treatment earlier than their sibling (sets 1 and 2) or for those who received idursulfase‐IT compared with their siblings who did not (sets 4 and 5). An exception to this pattern was seen in set 3: siblings had similar VABS‐II ABC scores at the same chronological age (Figure 3). All domains of VABS‐II (adaptive behavior, communication, daily living, motor, and socialization) showed a similar pattern to that of the composite VABS‐II ABC score (Figures 3 and S2).

## 4 DISCUSSION

The data presented here suggest an association between early initiation of treatment with IV idursulfase and idursulfase‐IT and higher cognitive functioning levels than those associated with later treatment initiation. Compared with their older siblings, the younger siblings tended to have higher levels of cognitive function at the beginning of the observation period and at the same chronological ages. Furthermore, idursulfase‐IT treatment appeared to be generally associated with stabilization of cognitive function compared with no idursulfase‐IT treatment in children younger than 13 years of age.

Comparison of data from sets of siblings with shared genotypes and similar environments can provide valuable information on the effects of treatment in MPS II. Indeed, data from the Hunter Outcome Survey (a global observational registry study of patients with MPS II) have suggested that the level of similarity in the clinical presentation of MPS II between male siblings is greater than in other lysosomal storage diseases, such as Fabry disease.^24^ In the present analysis, all sets of siblings had missense *IDS* variants (genotype was unavailable for one patient, but it was assumed that the patient had a missense variant based on sibling data); although many missense variants lead to non‐neuronopathic disease, there is a subset associated with the neuronopathic form of MPS II.^25^ While predicting a patient's phenotype from their genotype is challenging in MPS II,^25^ developmental insights (such as for cognitive decline) obtained from sibling studies remain important. Although these studies are limited by small sample sizes, sibling comparisons can be useful in providing a frame of reference for treatment response in MPS II.

A report of two siblings with neuronopathic MPS II illustrated the potential benefit of early IV ERT therapy, in which—in contrast with the older sibling—presymptomatic treatment allowed the younger sibling to remain virtually free from somatic symptoms.^26^ In a case series review of eight patients with MPS II who started IV idursulfase before 1 year of age (because of a family history of MPS II), improvements and/or stabilization of somatic disease were observed after treatment. In some cases, caregivers subjectively reported that the affected family members who initiated treatment at the earlier age had a less severe disease course than affected family members who initiated treatment at the older age.^27^ In the present analysis, in age‐matched assessments, younger siblings tended to have a higher DAS‐II GCA score before idursulfase‐IT treatment initiation than older siblings. In three of the sibling sets in which this was observed, the youngest sibling received IV idursulfase before 1 year of age, which may suggest that early IV ERT treatment is associated with a stabilization of cognitive function.

A further case report of three adult brothers with non‐neuronopathic MPS II demonstrated the need for early ERT treatment to optimize treatment outcomes. These patients, who were diagnosed in early adulthood, began IV idursulfase therapy aged 44–51 years. Although some subjective measures improved after 12 months of treatment (e.g., energy and exercise tolerance), objective measures showed only limited improvement.^28^

Data from individuals with MPS II have also been compared with healthy siblings, to assess the natural progression of the disease. In a case study of a twin who had received IV idursulfase from the age of 3 months, no clinical manifestations of MPS II had developed after 3 years of treatment. Notably, there were few differences when compared with his healthy twin brother (i.e. only mild deformity of one vertebra and a lower IQ in the sibling with MPS II). Thus, early initiation of treatment with IV idursulfase may have the potential to modify the progression of MPS II.^29^ It is generally agreed that IV idursulfase does not cross the blood–brain barrier at sufficient levels to reduce cognitive impairment. As such, it is likely that idursulfase‐IT—rather than IV idursulfase—is responsible for the higher cognitive function seen in patients who initiated ERT at a young age, when compared with patients who initiated ERT later in life.

There are the following limitations in this early treatment sibling comparison. No formal statistical analyses were performed, and the number of sibling sets is small. The siblings in our analyses may not be representative of all of the patients involved in the clinical trials or in real‐world scenarios. For example, this study only included patients with missense *IDS* variants, meaning that generalizations to other genetic variants cannot be made. The increased cognitive function observed in the younger siblings may be partly due to the earlier diagnosis and earlier initiation of IV idursulfase when compared with their older siblings. This might have contributed to improvement in the younger siblings' overall health by the mitigation of somatic disease manifestations. Earlier diagnosis in younger siblings may also have had other indirect impacts on cognitive function that were not explored in this study, with factors such as use of hearing aids, occupational, physical, and speech and language therapy, or medication for attentional difficulties being allowed during the study based on individual patient need and potentially influencing outcomes.

## 5 CONCLUSIONS

Our results suggest that early initiation of idursulfase‐IT may stabilize or slow cognitive decline in some patients with neuronopathic MPS II with missense *IDS* variants. However, there were some confounding factors, such as earlier diagnosis and earlier initiation of treatment with IV idursulfase in younger siblings when compared with older siblings. These data, taken together with the results from the phase 2/3 study, substudy, and its associated extension, suggest a potential cognitive benefit of early treatment in young patients with MPS II. Unfortunately, after many years of extensive review and regulatory discussions, the idursulfase‐IT data were found to be insufficient to meet the evidentiary standard to support regulatory filings for the use of idursulfase‐IT in the treatment of MPS II. Idursulfase‐IT will continue to be made available to patients who are currently enrolled in the ongoing open‐label extension studies until other treatment options are available to address the cognitive symptoms.

## AUTHOR CONTRIBUTIONS

JM, BKB, PH, LGGS, MRG, SAJ, NG, MIF, DB, and SR were involved in the acquisition of the data. MH, YW, KSY, DAHW, and DA were involved in the initial conception or design of the study. All authors contributed to the interpretation of the data, contributed to drafting of the manuscript, provided critical review during revisions, and approved the final manuscript for submission.

## FUNDING INFORMATION

The HGT‐HIT‐090, HGT‐HIT‐094, and SHP609‐302 studies were sponsored and funded by Shire (a Takeda Company). Under the direction of the authors, medical writing support was provided by Emma Davies PhD of Oxford PharmaGenesis, Oxford, UK, and was funded by Takeda Development Center Americas, Inc.

## CONFLICT OF INTEREST STATEMENT

JM has received consulting fees from and has participated on data safety monitoring boards or advisory boards for Denali Therapeutics, JCR Pharmaceuticals, REGENXBIO, Sanofi Genzyme, and Takeda; is a Principal Investigator for a post‐trial access program for intrathecal ERT for the neuronopathic form of MPS II (sponsored by Takeda), a phase 1/2 gene editing trial for adults with MPS II (sponsored by Sangamo Therapeutics), and phase 1/2 and phase 2/3 trials of IV ERT for MPS II (sponsored by Denali Therapeutics). BKB has received consulting fees from Aeglea, Agios, Alexion, AstraZeneca Rare Disease, Alltrna, BioMarin Pharmaceutical, Chiesi Farmaceutici, Horizon Therapeutics, JCR Pharmaceuticals, Moderna, Orchard Therapeutics, Passage Bio, PTC Therapeutics, Jnana Therapeutics, REGENXBIO, Sanofi Genzyme, Takeda, Travere, and Ultragenyx; has received payment or honoraria for lectures, presentations, speakers bureaus, manuscript writing or educational events from Alexion, BioMarin Pharmaceutical, Chiesi Farmaceutici, Horizon Therapeutics, Sanofi Genzyme, Takeda, and Ultragenyx; has participated on data safety monitoring boards or advisory boards for BioMarin Pharmaceutical, Freeline, JCR Pharmaceuticals, Moderna, and Takeda; has performed contracted research for Takeda and has been involved in company‐sponsored clinical trials with BioMarin Pharmaceutical, Denali Therapeutics, Homology Medicines, JCR Pharmaceuticals, Jnana Therapeutics, Sangamo Therapeutics, Synlogic, Takeda, and Ultragenyx. PH has received grants or contracts from BioMarin Pharmaceutical, Denali Therapeutics, Grace Science, Takeda, QED, Adrenas Therapeutics, Sangamo, Prevail/Lilly, Ascendis, ASPA, Idorsia, JCR Pharmaceuticals, Orphazyme, REGENXBIO, Sanofi Genzyme, Calcilytics, Immusoft, Allievex, Amicus Therapeutics, Azafaros and Homology. He has received consulting fees from Aeglea, BioTherapeutics, Audentes, AVROBIO, Capsida Biotherapeutics, Chiesi, Edigene, Grace Science, Inventiva Pharma, Neurogene, Novel Pharma, Orchard Therapeutics, Rallybio, Renoviron, Saliogen, and Sanofi Genzyme; and has received payment or honoraria for lectures, presentations, speakers bureaus, manuscript writing or educational events from BioMarin Pharmaceutical. LGGS has received consulting fees from BioMarin Pharmaceutical, Sanofi Genzyme, Takeda, and Ultragenyx Pharmaceutical; has received payment or honoraria for lectures, presentations, speakers bureaus, manuscript writing or educational events from BioMarin Pharmaceutical, Sanofi Genzyme, Takeda, and Ultragenyx Pharmaceutical, and has received research support from Takeda. MR‐G has received consulting fees, payment, or honoraria for lectures, presentations, speakers bureaus, manuscript writing or educational events, and research support from Takeda. SAJ has received grants or contracts from Orchard Therapeutics (SRAs and consulting plus investigator); has received consulting fees and payment or honoraria for lectures, presentations, speakers bureaus, manuscript writing or educational events from Alexion Pharmaceuticals, AVROBIO, BioMarin Pharmaceutical, Denali Therapeutics, Orchard Therapeutics, REGENXBIO, Sanofi Genzyme, Takeda, and Ultragenyx Pharmaceutical. He has received support for attending meetings and/or travel from Sanofi and has received research support from Takeda. NG has received research support from BioMarin Pharmaceutical, Chiesi, Sanofi Genzyme, Takeda, and Ultragenyx Pharmaceutical. MIF has performed contracted research for Takeda, Denali Therapeutics, Sanofi Genzyme, Ultragenyx Pharmaceutical, Passage Bio, Aeglea Biotherapeutics, PTC Therapeutics, and Vtesse (Mallinckrodt Pharmaceuticals), and has received the Canadian Institute of Health Research Strategy for Patient Oriented Research Innovative Clinical Trial Multi‐Year Grant; has received consulting fees from Sanofi Genzyme, Takeda, and Simon‐Kucher; has received payment or honoraria for lectures, presentations, speakers bureaus, manuscript writing or educational events from Horizon Therapeutics; has participated on a data safety monitoring board or advisory board for Alexion Pharmaceuticals, Cyclo Therapeutics, Horizon Therapeutics, Recordati Rare Diseases, Sanofi Genzyme, Takeda, and Ultragenyx Pharmaceutical; is a member of the medical advisory board for the Canadian MPS Society and Allied Diseases and is a member of the Garrod Association Guideline Committee. DB has received research support from Takeda. SR has received consulting fees from AVROBIO, BioMarin Pharmaceutical, Chiesi Farmaceutici, Takeda, and Ultragenyx; has received payment or honoraria for lectures, presentations, speakers bureaus, manuscript writing or educational events, payment for expert testimony, and support for attending meetings and/or travel from AVROBIO, BioMarin Pharmaceutical, Chiesi Farmaceutici, and Takeda. MH was an employee of Takeda Development Center Americas, Inc. at the time of this study; and has stock or stock options for Takeda Pharmaceuticals Company Limited. YW is an employee of Takeda Development Center Americas, Inc.; and has stock or stock options for Takeda Pharmaceuticals Company Limited. KSY was an employee of Takeda Development Center Americas, Inc. at the time of this study and of the writing of the manuscript (current affiliation is Alexion Pharmaceuticals, Inc., AstraZeneca Rare Disease, Boston, MA, USA) and has stock or stock options for Takeda Pharmaceuticals Company Limited and AstraZeneca. DAHW was an employee of Takeda Development Center Americas, Inc.; and was a stockholder of Takeda Pharmaceuticals Company Limited at the time of the study. DA was an employee of Takeda Development Center Americas, Inc. at the time of this study and of the writing of the manuscript (current affiliation is Merck, Boston, MA, USA) and has stock or stock options for Takeda Pharmaceuticals Company Limited.

## ETHICS APPROVAL

The studies were conducted in accordance with the Declaration of Helsinki and written informed consent was obtained from the parent(s) or legally authorized guardian(s); assent from the patient was also acquired.

## Supporting information


**DATA S1.** Supporting Information.

**FIGURE S1.** Individual profile plots of DAS‐II GCA school‐age battery standard scores and VABS‐II scores by chronological age for the excluded sibling set (natural history study only).
**FIGURE S2A.** Individual profile plots of VABS‐II domain scores by chronological age for all siblings sets: communication domain.
**FIGURE S2B.** Individual profile plots of VABS‐II domain scores by chronological age for all siblings sets: daily living skills.
**FIGURE S2C.** Individual profile plots of VABS‐II domain scores by chronological age for all siblings sets: motor skills.
**FIGURE S2D.** Individual profile plots of VABS‐II domain scores by chronological age for all siblings sets: socialization.

## Data Availability

The datasets, including redacted study protocol, redacted statistical analysis plan, and individual participants' data supporting the results reported in this article will be made available within 3 months from initial request to researchers who provide a methodologically sound proposal. The data will be provided after their de‐identification in compliance with applicable privacy laws, data protection, and requirements for consent and anonymization.
