# Supplementary material for: Evaluation of early treatment with intravenous idursulfase and intrathecal idursulfase‐IT on cognitive function in siblings with neuronopathic mucopolysaccharidosis II
Source: J Inherit Metab Dis. 2024 Sep 9;48(3):e12790. doi: 10.1002/jimd.12790 (PMC12041835; doi:10.1002/jimd.12790)
Supplement: Supplementary file 1 — DATA S1. Supporting Information. [file JIMD-48-0-s001.docx]

# Supplementary materials

**Evaluation of early treatment with intravenous idursulfase and intrathecal idursulfase-IT on cognitive function in siblings with neuronopathic mucopolysaccharidosis II**

Joseph Muenzer ꟾ Barbara K. Burton ꟾ Paul Harmatz ꟾ Luis González Gutiérrez-Solana ꟾ Matilde Ruiz-Garcia ꟾ Simon A. Jones ꟾ Nathalie Guffon ꟾ Michal Inbar-Feigenberg ꟾ Drago Bratkovic ꟾ Stewart Rust ꟾ Michael Hale ꟾ Yuna Wu ꟾ Karen S. Yee ꟾ David A.H. Whiteman ꟾ David Alexanderian

## **TABLE S1** Baseline characteristics and treatment status of the excluded sibling set (natural history study only).

## **FIGURE S1** Individual profile plots of DAS-II GCA school-age battery standard scores and VABS-II scores by chronological age for the excluded sibling set (natural history study only).

## **FIGURE S2A** Individual profile plots of VABS-II domain scores by chronological age for all siblings sets: communication domain.

## **FIGURE S2B** Individual profile plots of VABS-II domain scores by chronological age for all siblings sets: daily living skills.

## **FIGURE S2C** Individual profile plots of VABS-II domain scores by chronological age for all siblings sets: motor skills.

## **FIGURE S2D** Individual profile plots of VABS-II domain scores by chronological age for all siblings sets: socialization.

## **TABLE S1** Baseline characteristics and treatment status of the excluded sibling set (natural history study only).

|  | Younger sibling | Older sibling |
| --- | --- | --- |
| Age, years | 12.8 | 16.7 |
| Genotype | C.1122C>T | C.1122C>T |
| Variant category | Splice-site mutation | |
| Earliest available DAS-II GCA score (school-age battery)^a^ | 78 | 84 |
| Earliest available VABS-II ABC score | 90 | 87 |
| Age at initiation of IV idursulfase, years | 5.9 | 7.2 |
| Age at initiation of idursulfase-IT, years | No IT treatment | No IT treatment |

**^a^**Data collected at month 3 in the natural history study.
BSID-III, Bayley Scales of Infant and Toddler Development, Third Edition; DAS-II GCA, Differential Abilities Scales, Second Edition General Conceptual Ability; IT, intrathecal; VABS-II ABC, Vineland Adaptive Behavior Scales, Second Edition Adaptive Behavior Composite.

## **FIGURES**

##
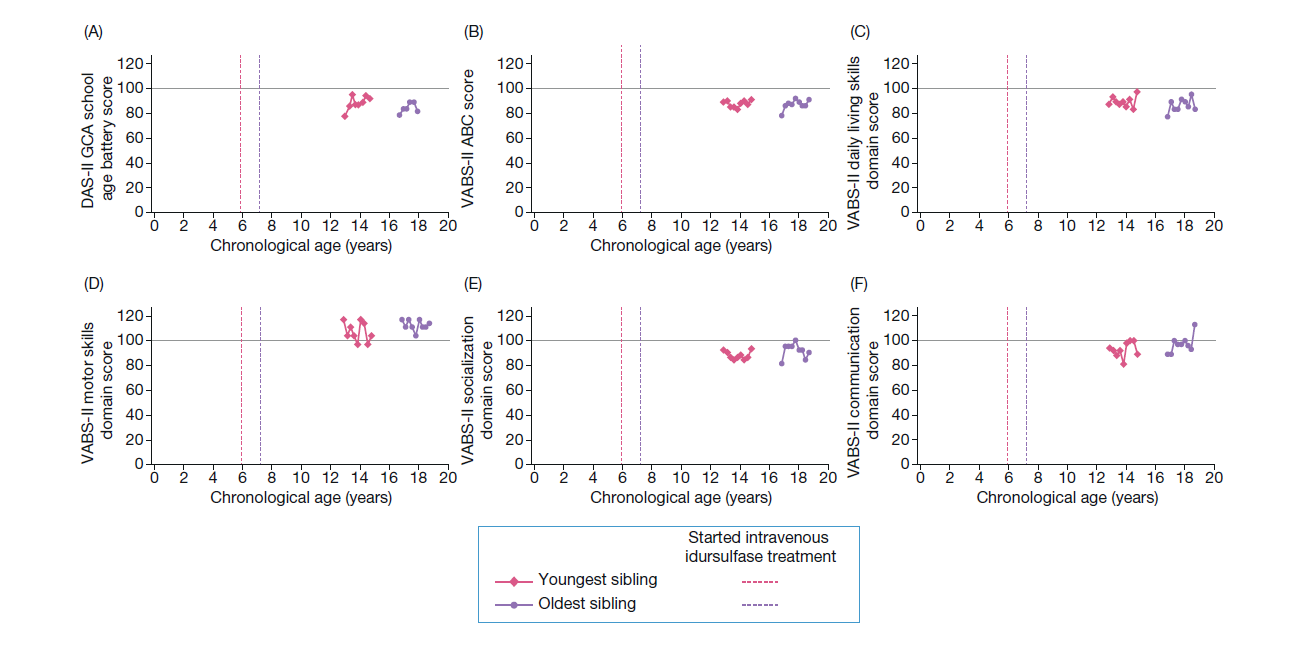
**FIGURE S1** Individual profile plots of DAS-II GCA school-age battery standard scores and VABS-II scores by chronological age for the excluded sibling set (natural history study only).

## ABC, Adaptive Behavior Composite; DAS-II GCA, Differential Abilities Scales, Second Edition General Conceptual Ability; VABS-II, Vineland Adaptive Behavior Scales, Second Edition.

##
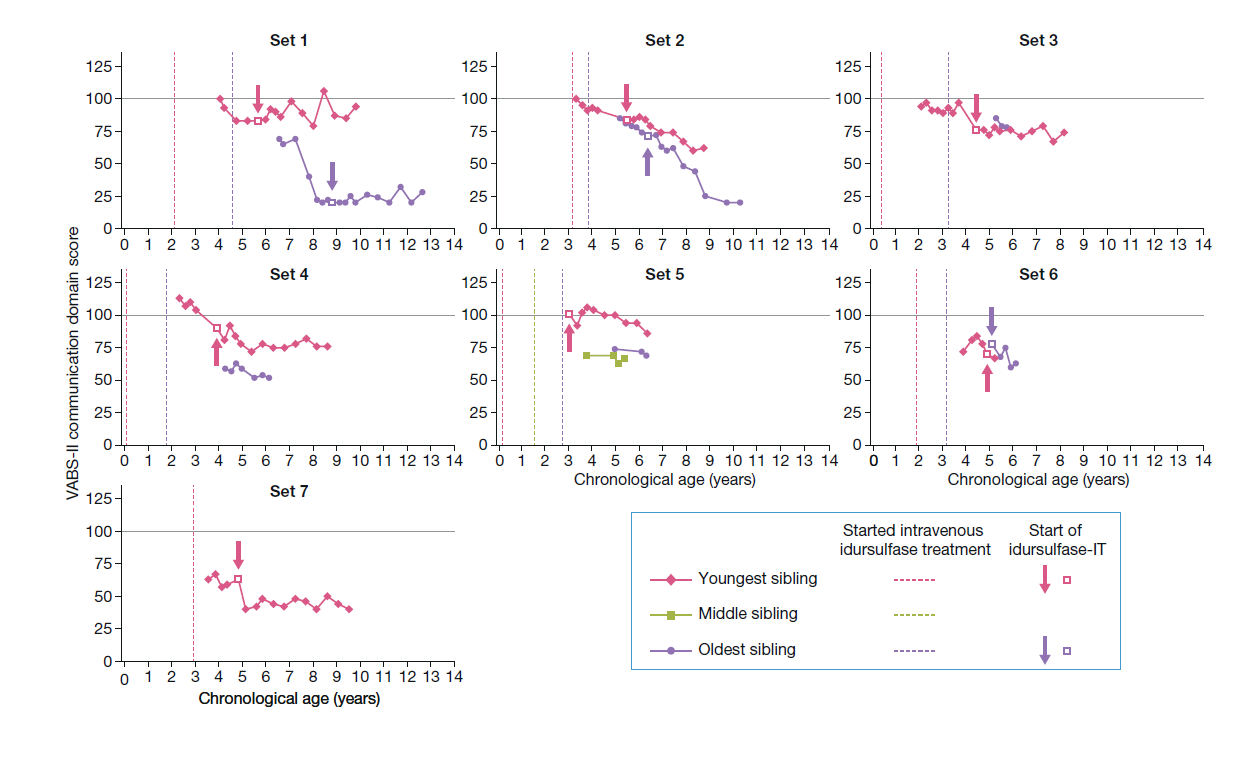
**FIGURE S2** Individual profile plots of VABS-II domain scores by chronological age for all siblings sets. (A) Communication domain, (B) Daily living skills, (C) Motor skills, and (D) Socialization.

(A)

##
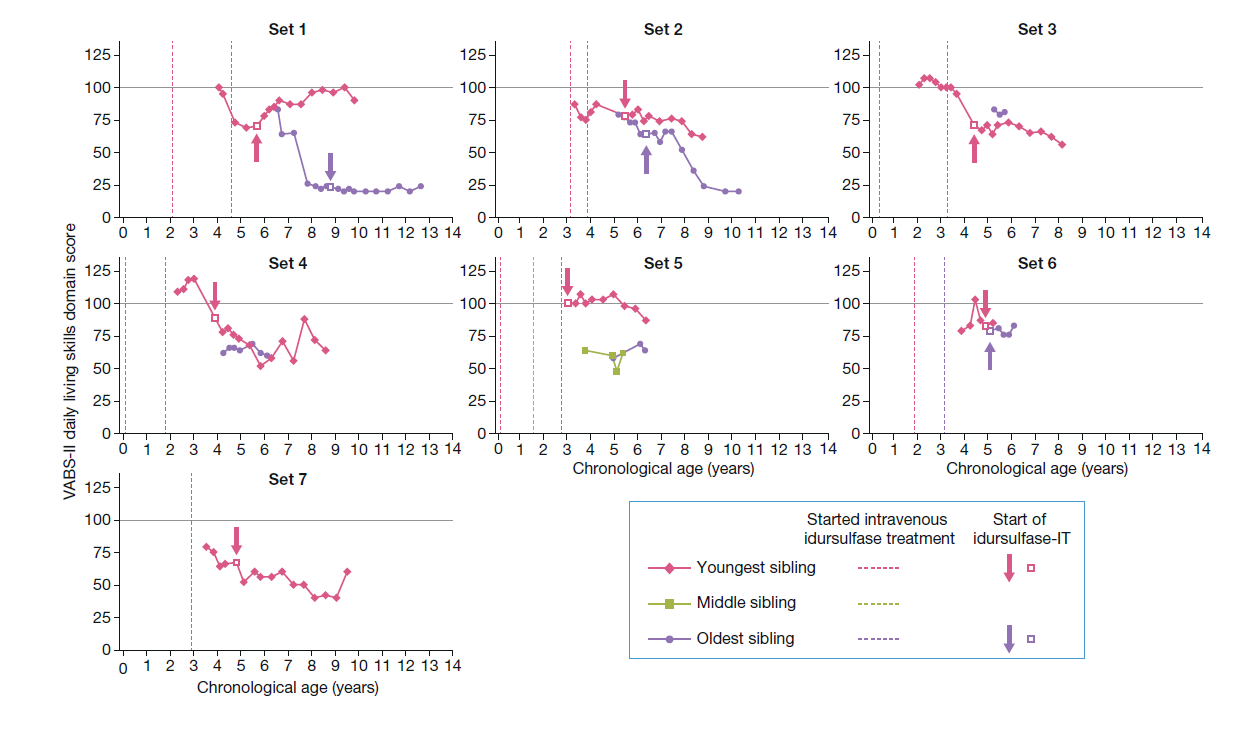
(B)

## (C)
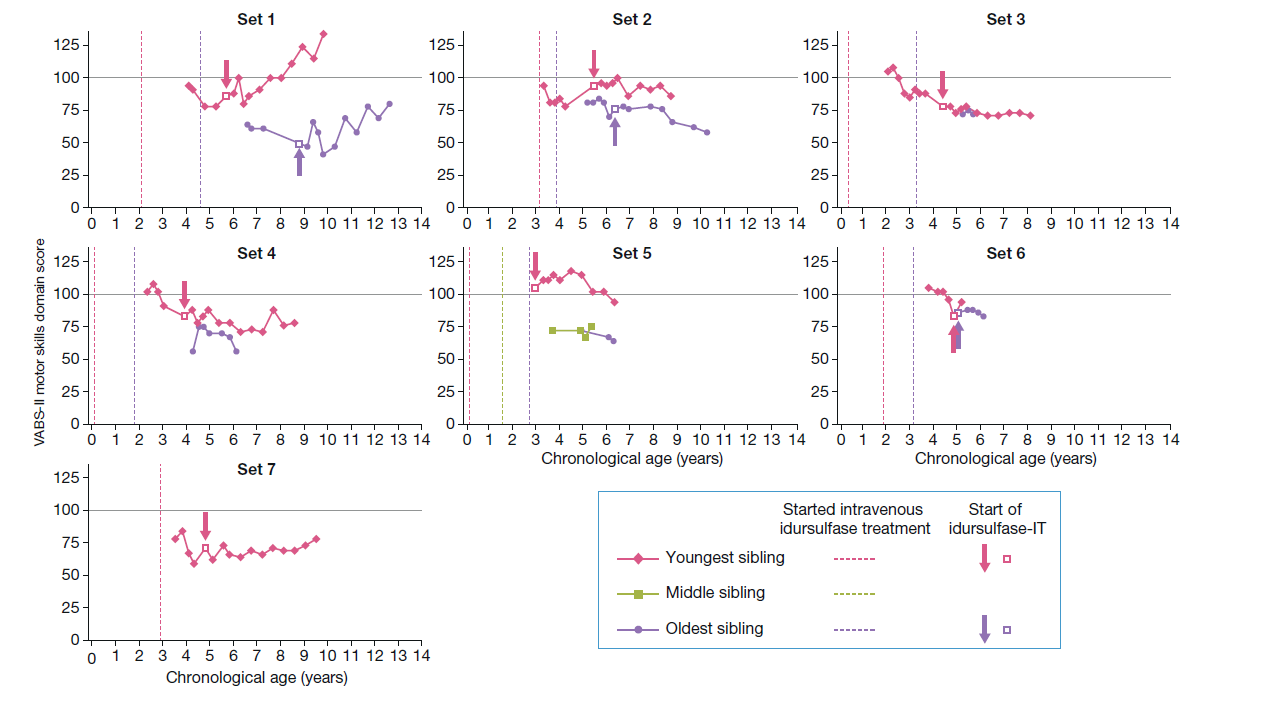


##
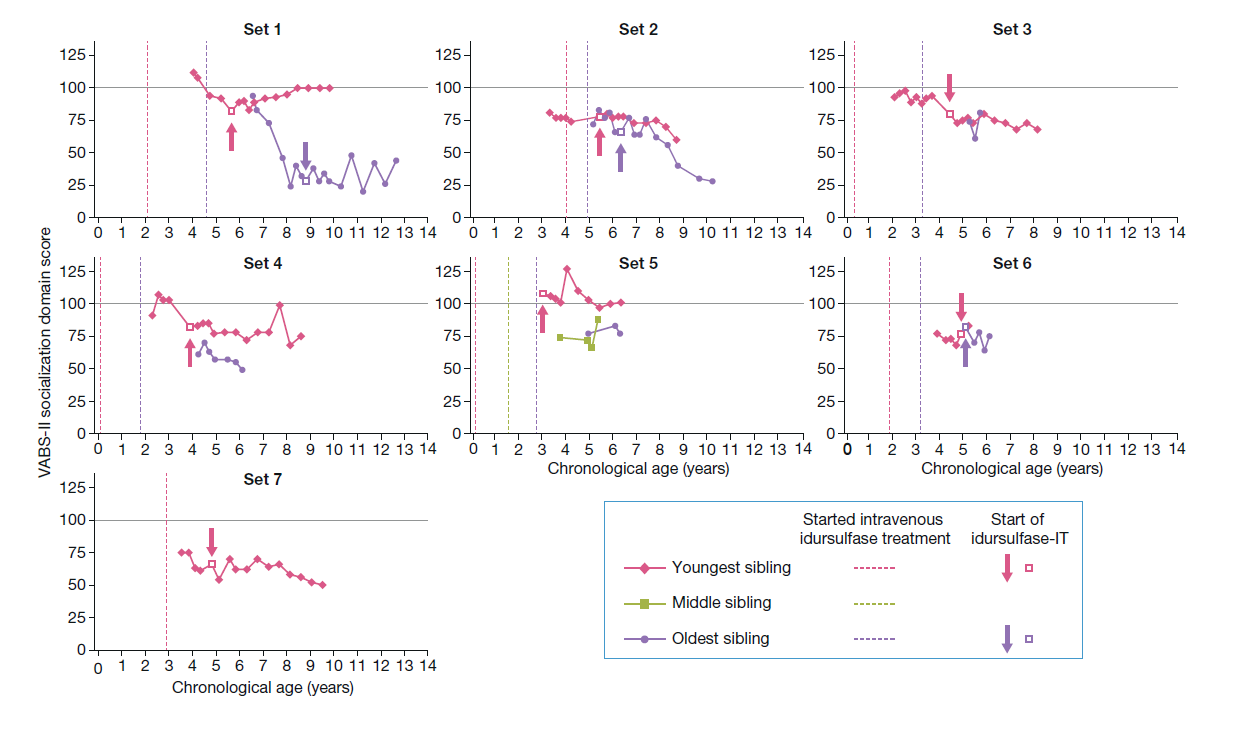
(D)

## IT, intrathecal; VABS-II, Vineland Adaptive Behavior Scales, Second Edition.
