# Supplementary material for: Evaluation of early treatment with intravenous idursulfase and intrathecal idursulfase‐IT on cognitive function in siblings with neuronopathic mucopolysaccharidosis II
Source: J Inherit Metab Dis. 2024 Sep 9;48(3):e12790. doi: 10.1002/jimd.12790 (PMC12041835; doi:10.1002/jimd.12790)
Supplement: Supplementary file 2 — FIGURE S1. Individual profile plots of DAS‐II GCA school‐age battery standard scores and VABS‐II scores by chronological age for the excluded sibling set (natural history study only). FIGURE S2A. Individual profile plots of VABS‐II domain scores by chronological age for all siblings sets: communication domain. FIGURE S2B. Individual profile plots of VABS‐II domain scores by chronological age for all siblings sets: daily living skills. FIGURE S2C. Individual profile plots of VABS‐II domain scores by chronological age for all siblings sets: motor skills. FIGURE S2D. Individual profile plots of VABS‐II domain scores by chronological age for all siblings sets: socialization. [file JIMD-48-0-s002.pdf]

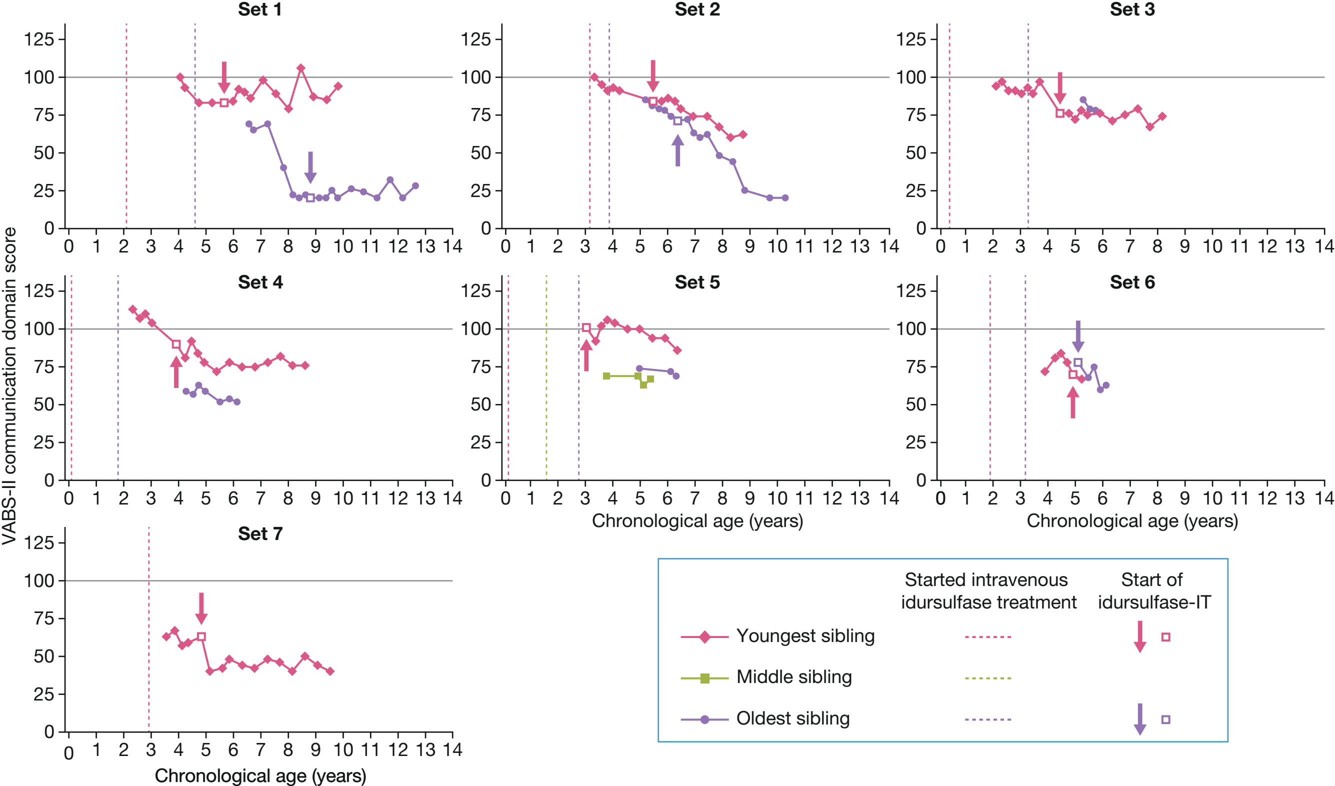
Set 1
Set 2
Set 3
Set 4
Set 5
Set 6
Set 7
VABS-II communication domain score
Chronological age (years)
Started intravenous idursulfase treatment
Start of idursulfase-IT
Youngest sibling
Middle sibling
Oldest sibling

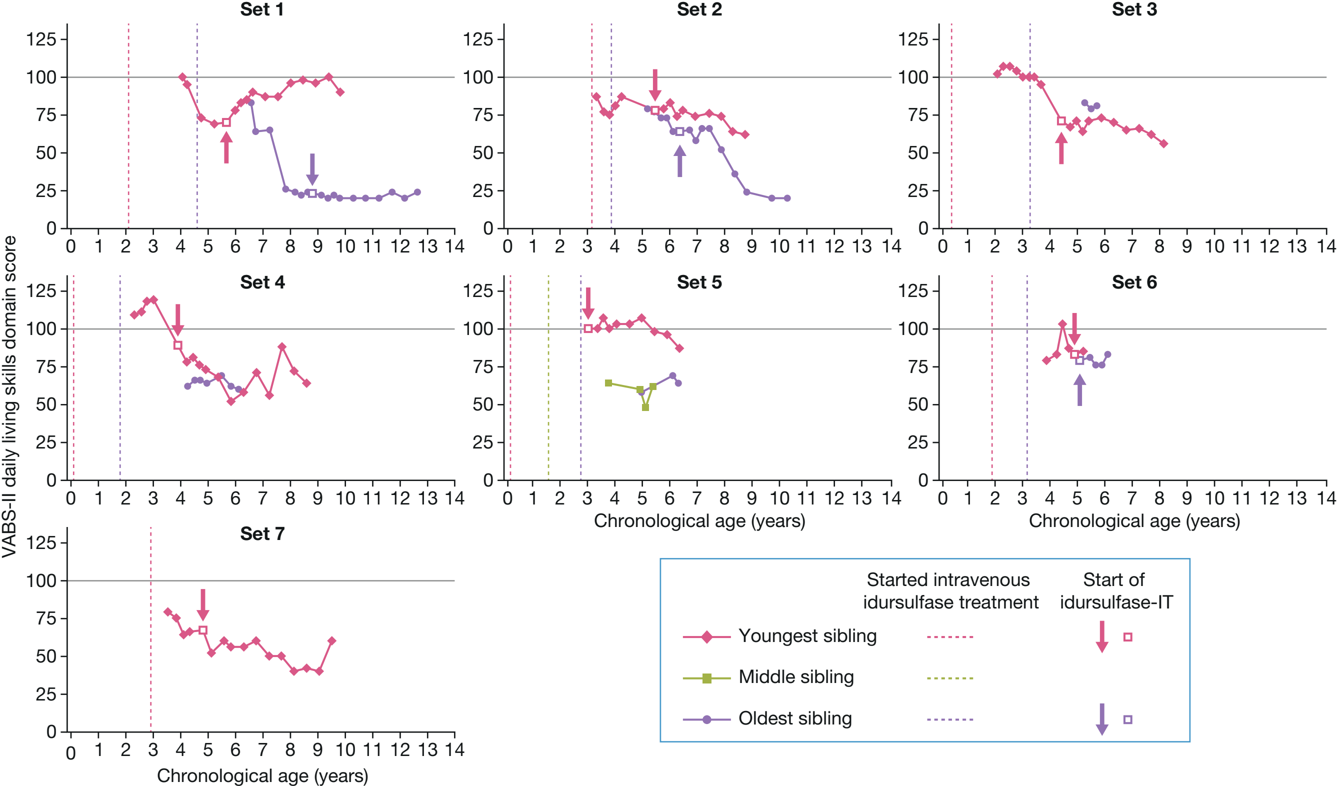

Set 1
Set 2
Set 3
Set 4
Set 5
Set 6
Set 7
VABS-II daily living skills domain score
Chronological age (years)
Started intravenous idursulfase treatment
Start of idursulfase-IT
Youngest sibling
Middle sibling
Oldest sibling

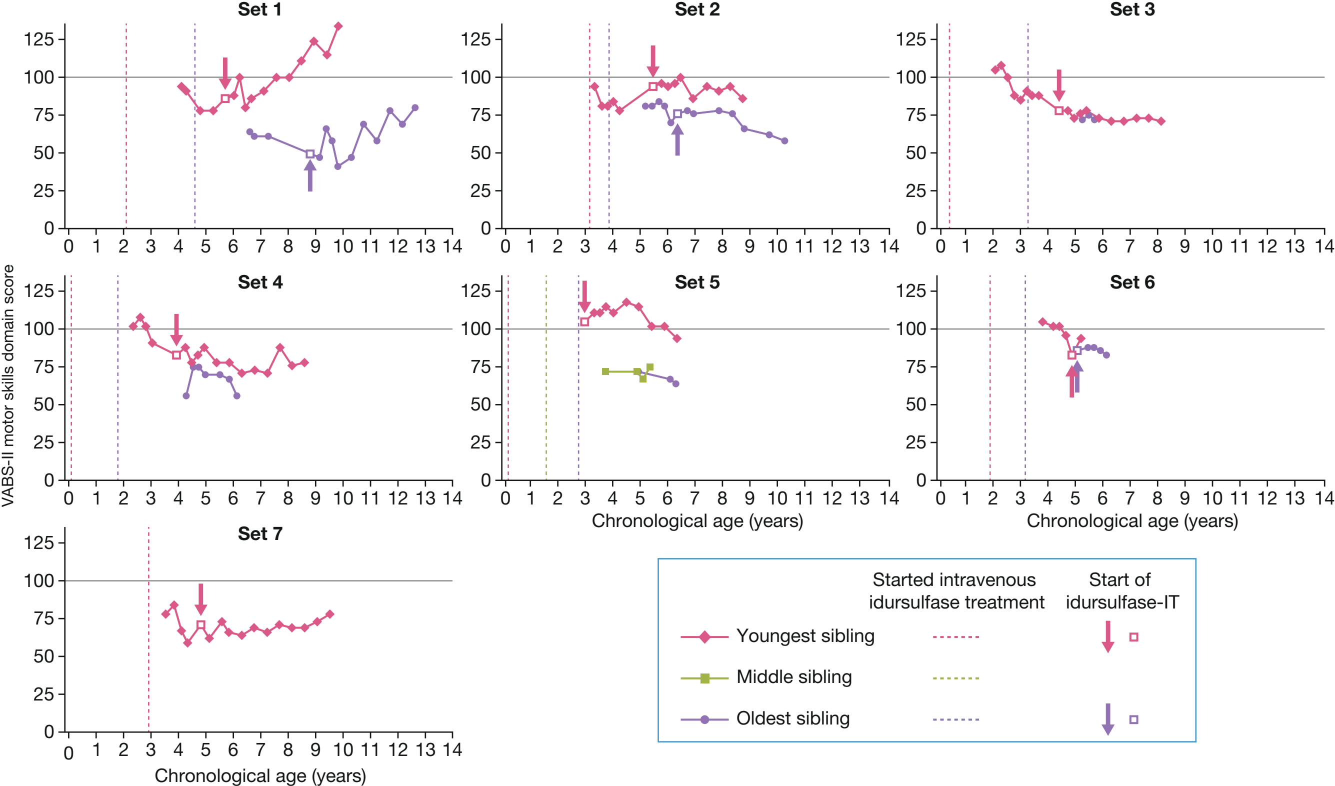
Set 1
Set 2
Set 3
Set 4
Set 5
Set 6
Set 7
VABS-II motor skills domain score
Chronological age (years)
Started intravenous idursulfase treatment
Start of idursulfase-IT
Youngest sibling
Middle sibling
Oldest sibling

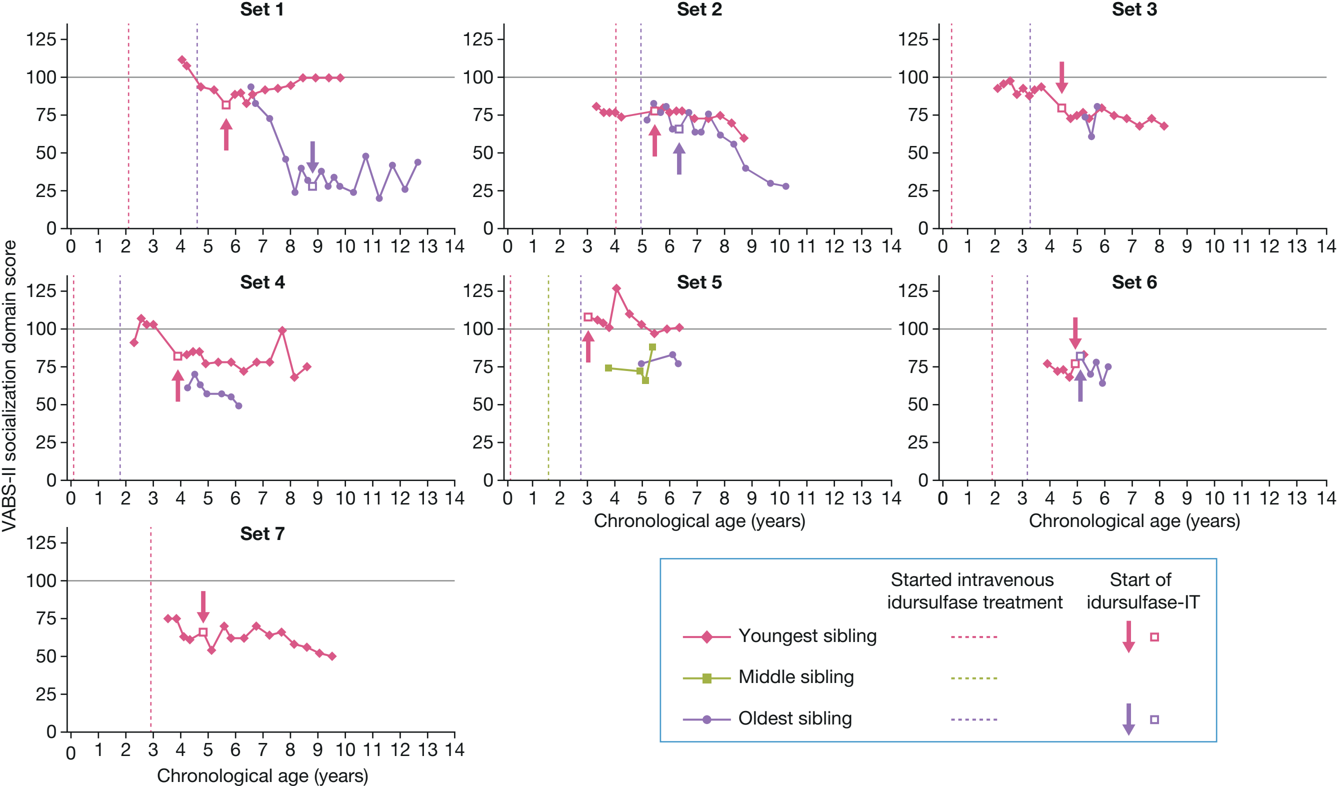
Set 1
Set 2
Set 3
Set 4
Set 5
Set 6
Set 7
VABS-II socialization domain score
Chronological age (years)
Started intravenous idursulfase treatment
Start of idursulfase-IT
Youngest sibling
Middle sibling
Oldest sibling

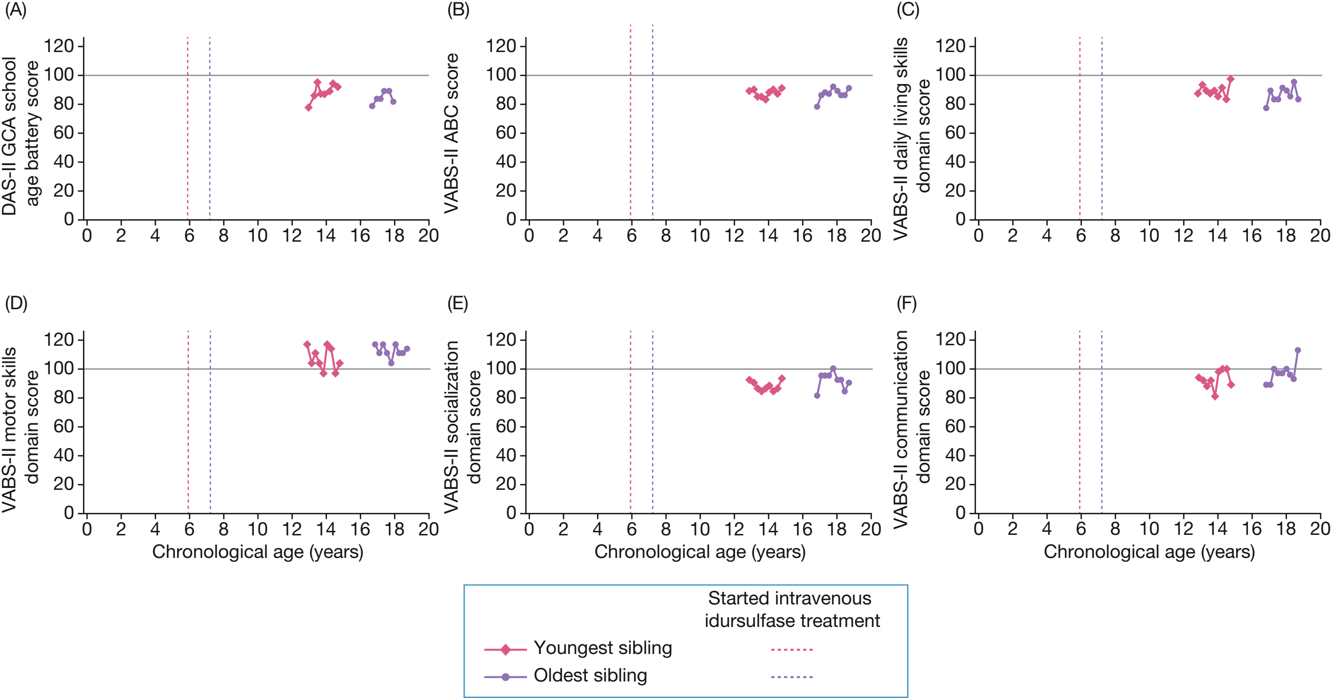

(A)
DAS-II GCA school age battery score
(B)
VABS-II ABC score
(C)
VABS-II daily living skills domain score
(D)
VABS-II motor skills domain score
(E)
VABS-II socialization domain score
(F)
VABS-II communication domain score
Chronological age (years)
Started intravenous idursulfase treatment
Youngest sibling
Oldest sibling
